# Characterization of Phenolic Compounds in Extra Virgin Olive Oil from Granada (Spain) and Evaluation of Its Neuroprotective Action

**DOI:** 10.3390/ijms25094878

**Published:** 2024-04-30

**Authors:** María Asunción López-Bascón, Inmaculada Moscoso-Ruiz, Rosa Quirantes-Piné, Raquel del Pino-García, Gloria López-Gámez, Andrea Justicia-Rueda, Vito Verardo, José L. Quiles

**Affiliations:** 1 Centro de Investigación y Desarrollo del Alimento Funcional (CIDAF), Avda. Del Conocimiento, 37, 18016 Granada, Spain; alopez@cidaf.es (M.A.L.-B.);; 2 Department of Analytical Chemistry, University of Granada, Avda. Fuentenueva S/N, 18071 Granada, Spain; rquirantes@cidaf.es; 3 Department of Nutrition and Food Science, Institute of Nutrition and Food Technology “José Mataix Verdú”, Biomedical Research Centre, University of Granada, 18071 Granada, Spain; 4 Department of Physiology, Institute of Nutrition and Food Technology “José Mataix Verdú”, Biomedical Research Centre, University of Granada, 18016 Armilla, Spain; 5 Research Group on Foods, Nutritional Biochemistry and Health, Universidad Europea del Atlántico, Isabel Torres, 21, 39011 Santander, Spain

**Keywords:** extra virgin olive oil, phenolic compounds, neuroprotective potential, anti-inflammatory potential, HPLC–MS, Protected Denomination of Origin

## Abstract

The olive oil sector is a fundamental food in the Mediterranean diet. It has been demonstrated that the consumption of extra virgin olive oil (EVOO) with a high content of phenolic compounds is beneficial in the prevention and/or treatment of many diseases. The main objective of this work was to study the relationship between the content of phenolic compounds and the in vitro neuroprotective and anti-inflammatory activity of EVOOs from two PDOs in the province of Granada. To this purpose, the amounts of phenolic compounds were determined by liquid chromatography coupled to mass spectrometry (HPLC–MS) and the inhibitory activity of acetylcholinesterase (AChE) and cyclooxygenase-2 (COX-2) enzymes by spectrophotometric and fluorimetric assays. The main families identified were phenolic alcohols, secoiridoids, lignans, flavonoids, and phenolic acids. The EVOO samples with the highest total concentration of compounds and the highest inhibitory activity belonged to the Picual and Manzanillo varieties. Statistical analysis showed a positive correlation between identified compounds and AChE and COX-2 inhibitory activity, except for lignans. These results confirm EVOO’s compounds possess neuroprotective potential.

## 1. Introduction

The agriculture of the Mediterranean countries is dominated by the cultivation of olive groves, whose star products are olive oil (OO) and table olives. Currently, more than 11.5 million hectares of olives are cultivated in more than 67 countries around the world, according to the International Olive Council (IOC) [1]. Most of this area (97%) is located in the Mediterranean countries, with Spanish OO accounting for 70% of European production and 45% of world production [2,3]. In Andalusia (Spain), more than 46% of the total agricultural area is dominated by olive cultivation, and specifically in Granada, more than 16% [4,5].

Within OOs, subtypes of virgin olive oils (VOOs) and extra virgin olive oils (EVOOs) are extracted by mechanical processes. Both VOO and EVOO contain 97–99% of lipids, mostly triglycerides, and 1–3% of minor components. This minor fraction includes more than 200 compounds, such as tocopherols, sterols, triterpenes, pigments, phenolics, and volatile compounds. In addition to providing aroma, taste, and color to the oil, the components of this minority fraction are responsible for most of its biological activities and functional properties, highlighting the phenolic compounds as the best known for their healthy potential [6,7,8]. Commission Regulation (EU) 432/2012 recognizes three health claims about minor components present in (E)VOO: vitamin E, mainly as α-tocopherol, phytosterols, and phenols. The health claim for phenols is related to the protection of blood lipids from oxidative stress, and it is attributable to OOs providing a minimum amount of 5 mg of hydroxytyrosol (Hyty), tyrosol (Ty), and derivatives per 20 g of product [9].

Among the beneficial properties of EVOOs, their phenolic compounds have been shown to have neuroprotective activity [10]. Neurodegenerative diseases are a current public health concern, especially because they occur in the elderly, and the worldwide population is getting older [11]. Neurodegenerative diseases include Alzheimer’s disease (AD), Parkinson, Huntington, Multiple Sclerosis and cerebral ischemia. In diseases such as AD or Parkinson, there is injury to brain neurons and subsequent destruction due to the appearance of insoluble extracellular deposits, amyloid plaques, formed by aggregation of the Aβ peptide. It has been shown that the formation of amyloid plaques triggers an inflammatory reaction in the brains of AD patients [12]. 

As there is no cure, treatment of neurodegenerative diseases currently focuses on slowing cognitive decline. Phenolic compounds have shown antioxidant power in experimental models of memory impairment, avoiding oxidative stress that may produce neurological damage and reducing the incidence of neurodegenerative disruption [13,14]. It has been demonstrated via in vitro, in vivo, and prevalence studies that oleuropein (Ol), Hyty, and oleocanthal, the main phenolic compounds of olive trees, may prevent neurodegenerative diseases such as AD or Parkinson by preventing the aggregation of Aβ peptides [15,16,17,18]. Among the proposed treatments, the inhibition of the activity of cyclooxygenase enzymes (COXs), especially COX-2, which is involved in inflammatory mechanisms caused by Aβ peptides [19,20], has been studied. Another mechanism of neuroprotective action to prevent AD that has been shown to be a useful therapeutic approach is the restoration of levels of acetylcholine (ACh) neurotransmitter via inhibition of acetylcholinesterase (AChE) and butyrylcholinesterase (BuChE). High levels of these enzymes reduce the levels of ACh in the synaptic gap, and consequently, an increase in AD progression occurs [21].

The geographical and genetic origin of the olives, the choice of agronomic practices, and the technological conditions of EVOO production determine the great variability in its phenolic composition [22]. Protected Designation of Origin (PDO) is a quality regime from the European Union for those products that have the strongest links to the place in which they are made [23]. In Granada, there are two PDOs for EVOO: *Poniente de Granada,* located in the west of Granada, with varieties Lucio, Hojiblanca, Picudo, and Picual; and *Montes de Granada,* distributed throughout the province, with the main varieties being Picual, Loaime, and Lucio [24,25]. EVOO from Granada has been widely studied in terms of antioxidant capacity, health-promoting properties, or bioavailability; however, there is a lack of information about the EVOO cataloged as PDO and its correlation with phenolic compounds, in addition to its neuroprotective potential.

The main objective of this research was to study the relationship between the concentration of phenolic compounds and the in vitro neuroprotective activity of EVOOs belonging to two PDOs in the province of Granada (Spain). For this purpose, the main phenolic compounds present in EVOO samples were quantified by HPLC–MS. Also, the neuroprotective effect of EVOO extracts was determined by evaluating their cholinergic and anti-inflammatory effects as inhibitors of AChE and COX-2, respectively.

## 2. Results and Discussion

### 2.1. Qualitative and Quantitative Characterization of EVOO Samples by LC-MS

The main phenolic acids in EVOO are hydroxybenzoic, *p*-coumaric, ferulic, gallic, syringic, vanillic, caffeic, o-coumaric, and sinapic acids; meanwhile, other types of polyphenols are flavonoids, lignans, secoiridoids, and phenolic alcohols [26,27]. Regarding flavonoids, highlight luteolin (Lut), apigenin (Api), and their derivatives; about lignans, pinoresinol (Pin) and acetoxypinoresinol (Ac-Pin) are the usual lignan contents in EVOO samples. Secoiridoids are phenolic compounds that are rarely found in nature; however, they appear in abundance in Oleaceae species. The main secoiridoids are methyloleuropein (Me-Ol), oleuropein (Ol), and ligstroside and their aglycones.

**Figure 1:**
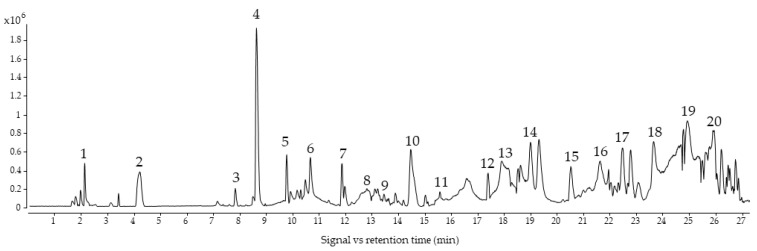
BPC (Base Peak Chromatogram) from EVOO sample pool. 1: QuiAc; 2: DiHyGli; 3: Van; 4: Hyty; 5: Ty; 6: EA 2; 7: DEDA; 8: HyDec Ol Ag 1; 9: Hy Ol Ag 1; 10: TMP-Ac; 11: Hyty-Ac; 12: Syr; 13: Pin; 14: Ac-Pin; 15: Hy-Ol-Ag 1; 16: Me-Ol-Ag 2; 17: Li-Ag 4; 18: Ol-Ag 1; 19: Hy-D-Ag 3; 20: Lut. Full name of abbreviations can be found in Table S1.

The base peak chromatogram from the EVOO sample pool is shown in Figure 1. The methodology proposed in this study allowed us to identify 42 compounds belonging to phenolic alcohols, secoiridoids, lignans, flavonoids, and phenolic acids. In some cases, several compounds with the same mass and molecular formula were detected, being isomers that could not be differentiated with the information obtained from their mass spectrum. Table S1 shows all identified compounds, including their abbreviations, retention times, m/z, molecular formulas, errors, and fragments.

After the identification, all samples of EVOO were quantified, obtaining the concentration of each compound (Table 1). As showed in Table 1, the compounds with the highest concentrations were elenolic acid (EA), with ranges between 6.9 (sample 13) to 80.4 (sample 12) µg g^−1^; hydroxydecarboxymethyl ligstroside aglycone (Hy-D-Li-Agl), ranged from 37 (sample 5) to 387 (sample 4) µg g^−1^; ligstroside aglycone (Li-Ag), from 20.29 (sample 13) to 448 µg g^−1^; and oleuropeine aglycone (Ol-Ag), with concentrations between 83.1 (sample 5) and 849 (sample 3) µg g^−1^, being the compound with higher concentration in all cases except for sample 2 (Picudo variety), whose majority phenolic compound is Li-Ag. These results agree with those of other authors who analyze EVOO samples from Italy and Spain. Figuereido-González et al. (2018) studied two different EVOO varieties from Spain, Cornicabra and Picual, whose main phenolic compounds quantified were Li-Ag, Ol Agl, and EA [28]. The same results were obtained by Criado Navarro et al. in 2020, when they analyzed 1245 samples of EVOO (varieties Picual, Arbequina, Hojiblanca, and Cornicabra), finding higher concentrations of Li-Ag and Ol-Agl than the rest of the phenolic compounds [29]. Fanali et al. (2018) found mainly secoiridoids in their 13 EVOO samples from Italy, showing major concentrations in Ol-Ag and Li-Agl [30]. Considering the sum of quantified compounds, samples 3, 6, and 9 stand out for having the highest total concentration, 1471, 1341, and 1245 µg of total phenolic compounds per gram of sample, respectively, which correspond to varieties Picual (samples 3 and 9) and Manzanillo (sample 6).

### 2.2. Determination of AChE Inhibitory Activity of EVOOs

The percentage of AChE inhibitory activity (2.5 mU) obtained when the bioactive compounds extracted from 0.150 g of EVOO are present in 250 μL of medium reaction are presented in Table 2. The ranges varied between 20.7 and 86.7% of inhibition. The highest inhibitory activity was obtained in samples 4, 6, 7, and 9, followed by 1, 2, 3, and 8, all of them from PDO *Poniente de Granada*, except samples 3 and 9, which are from PDO *Montes de Granada*. Regarding the variety of the samples, there does not seem to be any correlation between this factor and the AChE inhibitory activity. All EVOO excepting 13, 14, and 15 showed higher AChE inhibitory activity than 0.025 µM of physostigmin, a drug that prevents the destruction of acetylcholine and causes an indirect parasympathomimetic effect.

There are several works that have tested via in vitro assays the capacity to inhibit AChE of food or plants (edible or not) with the objective of avoiding the development of AD. Most of these products are fruits, such as apple [31], avocado [32,33], sweet cherry [34], pomegranate [35], pear [36], artichoke [37], or cochayuyo [38]. All of these studies attribute the power of anti-AChE to the content of phenolic compounds in the different matrices. 

Regarding oil, there are some authors that have evaluated different kinds of oils with the power of AChE inhibition, such as essential oils [39], hemp oil [40], coconut oil [41], or olive oil [42]. It is well known that EVOO has the potential to be a source of bioactive compounds. However, as far as we know, only a few works have studied in the last decade the effects of EVOO by studying the AChE inhibition. Amel et al. (2016) studied by in vivo models the effect of EVOO administered orally in mice and concluded that, with a dose of 5 mg kg^−1^ of body weight, EVOO provided protection against neurological affections and could be explained by its antioxidant composition, rich in phenolic compounds [43]. Additionally, Figuereido-González et al. (2018) essayed Cornicabra and Picual EVOO varieties via in vitro essays. They concluded that the Cornicabra variety showed better AChE inhibitory activity than Picual, although both of them demonstrated inhibition of AChE [28], which is in agreement with the presented results, as all analyzed EVOO samples showed inhibition of AChE. In the same year, Figuereido-González et al. investigated Mansa and Brava varieties of EVOO from Galicia (Spain) and reported that only the Brava variety inhibited AChE in a dose-dependent manner [44]. In this sense, they concluded that rich phenolic extracts from Galician EVOOs could act as neuroprotectors. However, Collado González et al. (2017) studied EVOO samples from Murcia (Spain), whose varieties were Arbequina, Cornicabra, Picual, Hojiblanca, and Cuquillo, and did not find any neuroprotective action [45]. There are other papers that correlate EVOO with its neuroprotective action, although using different strategies than AChE inhibition. All the works assign the power of AChE inhibition to the phenolic compound composition and content and study the influence of a single phenolic compound (such as Ty, Hyty, or Ol) on the AChE enzyme [46]. In our study, some individual phenolic compounds were tested to calculate their IC_50_, such as Ol, Lut, and Hyty, which gave values of 417 µM, 145 µM, and 328 µM, respectively. 

### 2.3. Determination of COX-2 Inhibitory Activity of EVOOs

Table 2 shows the inhibition percentages on COX-2 activity obtained when the bioactive compounds extracted from 0.625 mg of EVOO are present in 100 μL of medium reaction. In general, all samples had similar COX-2 inhibitory activity values, with ranges between 53% and 68% in all cases (Table 2). The samples 6, 7, 8, 9, and 15 showed the highest COX-2 inhibitory activity, while the samples 5 and 10 had the lowest. According to the obtained results, there does not seem to be a correlation between DPO and/or the variety of EVOO and the COX-2 inhibitory activity. Some authors, such as Cuffaro et al. (2023), obtained inhibition percentages of around 10% in EVOO from varieties Moraiolo, Frantoio, and Leccino (Italy) when tests were performed at 225 µg/mL of EVOO extracts [47]. On the other hand, when measuring COX-2 expression after EVOO consumption, unexpected results were achieved by Lakhder et al. (2016) when they determined the COX-2 content of serum rats after they were fed with EVOO at different concentrations in an in vivo study. They obtained more COX-2 in serum in rats that had consumed more EVOO, suggesting that the more the consumption, the more the expression of COX-2 [48]. However, the ability of EVOO polyphenols to inhibit the activity of COX-2 could counteract the pro-inflammatory effects of the higher concentration of this enzyme in serum. Since different effects of EVOO have been found in modulating the activity or expression of COX-2, more studies are required to clarify the pro- or anti-inflammatory effects that its consumption may have.

Literature shows that phenolic compounds from EVOO inhibit the action of inflammatory COX-2. In our study, some of them, such as Ol and Hyty, showed an IC_50_ of 9.65 µM and 10.8 µM, respectively, being 0.210 µM that of Celecoxib. In fact, PREDIMED (PREvencion con DIeta MEDiterranea, in English “Prevention with Mediterranean Diet”) study has demonstrated with a randomized trial the long-term effects of Mediterranean Diet on clinic events of cardiovascular diseases and showed via nutrigenomic studies the beneficial effects in health of several genetic variants of olives, including the inhibition of COX-2, among others [49,50]. Several studies have demonstrated the effect of specific phenolics on inflammation processes. Hyty and Hyty-Ac have been demonstrated to reduce the proinflammatory mediator in studies about rheumatoid arthritis involving this enzyme, among others [51]; methyl-oleocanthal was explored as a metabolite of EVOO and showed that it had the ability to decrease the overexpression of proinflammatory enzymes such as COX-2 [52]. Likewise, Cuffaro et al. (2023) showed that EVOO enriched with Ol from extract leaves offered more major anti-inflammatory properties than EVOO, as seen by the COX-2 inhibition [47]. Additionally, Carpi et al. demonstrated in 2019 that Ol and Oleacin reduced inflammation-related genes in adipocytes [53]. Regarding neuroprotective action, 2001 was the first time that it was demonstrated in vivo that inhibition of the cyclooxygenase isoenzymes COX-1 and COX-2 offered protection from Parkinson’s disease [54]. However, there are no studies about phenolic compounds from EVOO and inhibition of COX-2 focused on neuroprotection.

### 2.4. Correlation Analysis between Phenolic Composition and Neuroprotective/Antiinflammatory Activity of EVOOs

The correlation between the concentration of phenolic compounds and the neuroprotective and anti-inflammatory activity of EVOOs was evaluated, as several works assign the power of inhibition of AChE and COX-2 to the content of this type of substance. The objective of the activity was to establish if the neuroprotective and anti-inflammatory activities were linked to the concentration of phenolic compounds. For that purpose, a Pearson correlation analysis was carried out, which calculates the correlations between pairs of quantitative variables.

The correlation was made with the concentrations obtained of the quantified compounds and the percentage inhibitory activity of AChE and COX-2. Table 3 shows the compounds that have demonstrated a statistically significant correlation with the percentage inhibition of AChE and COX-2, respectively.

The correlations between the quantified compounds and the AChE inhibitory activity (Table 3) are positive; that is, the higher the concentration, the greater the inhibitory activity. For example, in sample 6, the compound Li-Ag 4 has a concentration of 348 μg g^−1^, which inhibits AChE enzymatic activity by 87%. The highest value in concentration offers the highest value of inhibitory activity. Similar to the behavior with the inhibitory activity of AChE, the correlations between the quantified phenolic compounds and the inhibitory activity of COX-2 are in general positive; that is, the higher the concentration, the greater the inhibitory activity. For example, in sample 9, the compound Ol Ag 2 has a concentration of 569 μg g^−1^, and such a sample inhibits COX-2 enzymatic activity by 68%. These results are consistent with scientific literature, as there are several studies that have found neuroprotective and antiinflammatory properties in compounds from EVOO [54,55]. Furthermore, the predicted intestinal absorption of the main phenolic compounds found in EVOO samples has been demonstrated to be higher than 30%, which is considered to be highly absorbed when such compounds are orally administered (http://biosig.unimelb.edu.au/pkcsm/prediction, accessed on 12 March 2024).

However, concentrations of lignan compounds (Ac-Pin, Syr, and Pin) present a negative correlation with COX-2 inhibitory activity, with the lowest *p*-values (Table 3). This means that the lower the lignan compound, the higher the COX-2 inhibition. These results do not agree with what has been found in the literature. In their review, Young Jang et al. (2022) compile several pieces of data from works that have studied lignan behavior as anti-inflammatory compounds [56]. Among the papers studied, they found that dietary lignans and their metabolites control the inflammatory response via suppression of different inflammatory pathways, as well as reduce inflammation by attenuating cytokine expression in in vivo studies. Regarding specific compounds, Pin has been shown to inhibit the COX-2 enzyme in in vitro studies, including studies that involve inhibition of cancer cell proliferation [57,58,59]. On the other hand, Syr has been demonstrated to regulate the inflammatory response by modulating the secretion and expression level of certain cytokines and thus attenuating inflammation from diseases such as osteoarthritis, lung inflammation, or cardiac dysfunction [60,61,62]. Our differences with the existing studies can be due to our in vitro approach and the small sample size. It should take a larger number of sample analyses to achieve a statistically significant correlation. Furthermore, it is important to note that biological activity does not depend only on the individual compounds but also on synergistic or antagonistic effects, on which there is a lack of scientific information.

According to the methodology used, the samples that presented the highest AChE inhibitory activity were 4, 6, 7, and 9, while the samples that presented the highest COX-2 inhibitory activity were samples 6 and 9. Thus, samples 6 and 9 presented the highest inhibitory potential (AChE and COX-2) and a high total concentration of phenolic compounds, 1341 and 1245 μg g^−1^, respectively. However, sample 3 has the highest total concentration of compounds (1471 μg g^−1^), but its COX-2 inhibitory activity does not stand out from most samples.

### 2.5. Similarities and Differences among EVOO Analyzed

A comparative analysis among EVOOs was made according to genetic varieties and geographic situation (in terms of PDO). To our knowledge, there is little available literature to determine the importance of genetic factors in phenolic variability between olive trees, but these studies suggest that this genetic factor could explain 60–80% of total variability between species [63]. 

**Figure 2:**
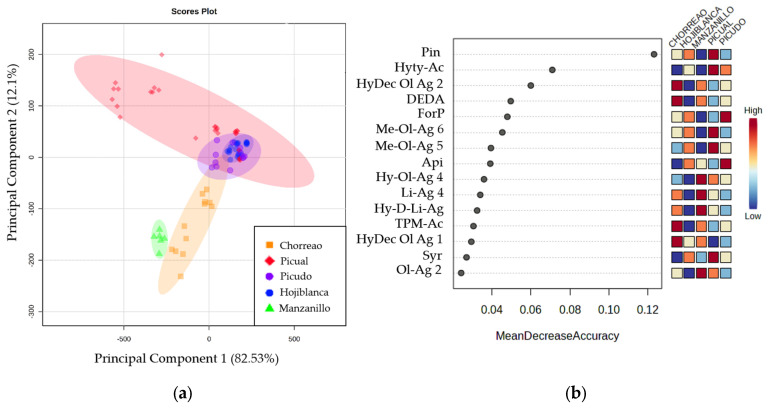
(**a**) Two-dimensional Principal Component Analysis plot obtained from studying the compounds quantified in the EVOOs of different varieties: Chorreao (orange), Hojiblanca (blue), Manzanillo (green), Picual (red), and Picudo (purple). (**b**) Random Forest plot that shows the quantified compounds with the greatest discriminating power between the varieties studied: Chorreao, Hojiblanca, Manzanillo, Picual, and Picudo.

For that reason, it was decided to study differences between EVOOs according to the variety of their origin. Figure 2a shows the 2-dimensional Principal Component Analysis (PCA) plot, which explains 94.4% of total variability, with principal component 1 explaining up to 82.3% of the variability.

A clear separation was found among the varieties Chorreao and Manzanillo and a group of samples of the Picual variety. However, the rest of the samples of the Picual, Hojiblanca, and Picudo varieties showed similarities between them, as they were grouped in the center of the graph. Furthermore, with the objective of studying the discriminatory power among varieties of quantified compounds, Random Forest analysis was applied. The ranking according to discrimination accuracy is shown in Figure 2b, where Pin and Hyty-Ac were the compounds with the highest discriminatory power among the varieties studied. 

**Figure 3:**
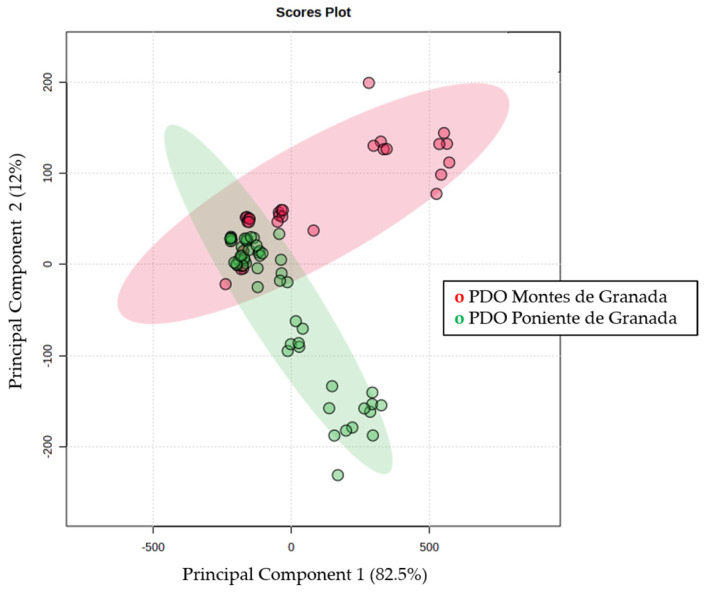
Two −dimensional Principal Component Analysis plot obtained by evaluating quantified compounds from PDO *Montes de Granada* (red) and *Poniente de Granada* (green).

Figure 3 shows the 2-dimensional PCA plot that explains 94% of the total variability, with principal component 1 explaining up to 82.5% of the variability. There is a clear separation between the two PDOs; however, a group of samples overlaps. The different designations of POD explain different geographical and climatological distributions, which can give rise to a clear separation. On the contrary, EVOOS obtained from olives close to each other will share many characteristics.

Table 4 shows the 23 compounds that showed significant differences between the PDO *Poniente de Granada* and the PDO *Montes de Granada*. Among them, phenolic alcohols and secoiridoids stand out. In contrast, the inhibition of AChE and COX-2 showed no significant differences between the PDOs. Some authors have studied the composition of phenolic compounds from different EVOO varieties and PDO. López-Huertas et al. studied in 2021 the differences between the content of specific phenols in EVOO from Spain depending on the stage of maduration, whose varieties in common with this study were Hojiblanca, Picudo, and Picual [64]. They showed great variability in the polyphenol concentration based on the stage of ripening. Picual variety concentrations vary between 232.8 and 317 mg kg^−1^; Hojiblanca between 83.3 and 214.5 mg kg^−1^; and Picudo between 75.3 and 265.7 mg kg^−1^. These results agree with our work; our Picual samples had a concentration of polyphenols ranging between 239 and 1471 mg kg^−1^, Hojiblanca between 263 and 579 mg kg^−1^, and Picudo between 369 and 619 mg kg^−1^. In all cases, in our study, higher amounts of phenolic compounds have been quantified. Other work from 2018 evaluated 50 EVOOs catalogued in nine different PDOs, including *Poniente de Granada*, with varieties such as Picudo, Hojiblanca, and Picual, among others [65]. The mean total phenolic compounds in Picudo were 101.8 mg kg^−1^, in Hojiblanca 104.8 mg kg^−1^, and in Picual 124.7 mg kg^−1^. Without exception, the total phenolic compounds in the samples analyzed in our study were higher. However, to our knowledge, there is a lack of scientific literature about specific phenolic composition differentiation between varieties and PDOs. This may be because there are no legal limits on phenolic compound composition established by EU regulation, and an oil can be classified as EVOO irrespective of its content of phenolic compounds [66]. Despite the significant differences observed between the two PDOs in relation to the phenolic compounds analyzed, there is a great deal of variability between samples. Before reaching reliable conclusions about POD, a more in-depth study should be carried out to validate the results by increasing the sample size.

## 3. Materials and Methods

### 3.1. Samples

The EVOO samples studied were from the province of Granada (Spain). They included 15 samples whose varieties were Hojiblanca (4 samples), Picudo (2), Picual (5), Chorreao (2), Manzanillo (1), and a coupage (mix of Picual, Arbequino, and Hojiblanca). The samples were supplied by different mills and cooperatives in the province.

### 3.2. Reagents and Equipment

All chemicals used were of analytical grade. LC-MS-grade methanol (MeOH) and acetic acid from Fisher-Scientific (Madrid, Spain) were used for extraction and to prepare mobile phases. Deionized water (18 MΩ·cm) supplied by a Milli-Q water purification system from Millipore (Bedford, MA, USA) was used to prepare the chromatographic aqueous phase (phase A). HPLC-grade *n*-hexane was purchased from Panreac (Barcelona, Spain). For the quantification of phenolic compounds, the following analytical standards were used: Ty, vanillic acid, luteolin, apigenin, *p*-coumaric acid, ferulic acid, and quinic acid were purchased from Sigma Aldrich (St. Louis, MO, USA), HYTY was acquired from Cayman (Ann Arbor, MI, USA), and oleuropein was purchased from Extrasynthese (Lyon, France).

For the COX-2 assay, a COX-2 Inhibitor Screening Kit from Merck (Madrid, Spain) was purchased. For the analysis of AChE inhibition, acetylcholinesterase from *Electrophorus electricus* (lyophilized powder, 500 U), Base Trizma^®^ (99.9% purity), chloridric acid (37%), Ellman Reactive (5,5′-dithiobis-(2-nitrobenzoic) acid, DTNB, 98% purity), acetylthiocholine iodide (≥98% purity), and physostigmine salicylate were purchased from Merck (Madrid, Spain). In both assays, a spectrophotometer from BioTek Synergy from Agilent Technologies (Santa Clara, CA, USA) was used.

Chromatographic separation for the identification of phenolic compounds was carried out in an Agilent 1260 HPLC System (Agilent Technologies, Palo Alto, CA, USA) by using a Zorbax Eclipse Plus RP-C18 (150 mm × 4.6 mm i.d., 1.8 μm). Q-TOF mass spectrometer equipped with an ESI Jet Stream interface (model 6540 from Agilent Technologies) operating in negative ion mode. Data from HPLC-ESI-QTOF MS/MS was treated with the software MassHunter (version B.06.00, Agilent Technologies). Quantification of phenolic compounds was performed with HPLC-TOF-MS using RRLC 1200 chromatography equipment (Agilent Technologies, Palo Alto, CA, USA). The column was the same as for the identification. The chromatograph was coupled to a microTOF system from Bruker Darlonik GmbH (Bremen, Germany) equipped with an ESI interface model G1607 (Agilent Technologies). Data from HPLC-TOF were treated with DataAnalysis 4.0 and TargetAnalysis 1.2 (Bruker Daltonics).

Other laboratory equipment was a centrifuge model Sorvall™ ST 16R and the Eppendorf™ Concentrator Plus, both from Thermo Fisher Scientific (Waltham, MA, USA).

### 3.3. Sample Preparation

Phenolic compounds were extracted, in duplicate, from EVOO samples according to the methodology proposed by López-Huertas et al. and Ballus et al. [64,67] with modifications. Briefly, 2.5 g of EVOO were weighed and dissolved in 10 mL of n-hexane, mixing for 1 min. The extraction was carried out with 10 mL of MeOH:H_2_O (60:40, *v*/*v*). The mixture was vigorously shaken with a vortex for 2 min and centrifuged at 1200× *g* for 10 min at 10 °C, and the polar fraction was separated. This process was repeated three times for each sample, and the polar fractions were collected and evaporated under vacuum in speed-vac at a temperature below 40 °C. The obtained residue was dissolved in 250 µL of MeOH:H_2_O (50:50, *v*/*v*) and centrifuged at 12,100× *g* at 10 °C for 10 min. Extracts were conserved at −20 °C until analysis.

### 3.4. LC−MS Analysis of Phenolic Compounds

The procedure used has been described in detail in previous reports [64,67]. For identification and quantification, the chromatographic methodology was the same. Mobile phase A was water with 0.25% acetic acid, and mobile phase B was MeOH. The chromatographic method was as follows: 0 min, 5% B; 7 min, 35% B; 12 min, 45% B; 17 min, 50% B; 22 min, 60% B; 25 min, 95% B; 27 min, 5% B; and finally a conditioning cycle of 7 min with the same conditions for the next analysis (total run: 34 min). The injected volume was 10 μL, and the injector needle was washed between injections with MeOH to avoid cross-contamination. The flow rate was 0.8 mL min^−1^, and the temperature of the column was maintained at 25 °C.

#### 3.4.1. Identification of Phenolic Compounds by LC−MS/MS

For identification of the main phenolic compounds, the sample extracts were analyzed by LC−QTOF MS/MS. Q−TOF analysis was performed in negative mode, with a mass range for compounds of 50–1700 m/z. Ultrapure N_2_ was used as ionization and drying gas, with temperatures of 325 °C and 400 °C and flow rates of 10 and 12 L min^−1^, respectively. The optimum values of source parameters were capillary voltage of 4 kV; nebulizing gas pressure of 20 psig; Q1 tension of 130 V; nozzle voltage of 500 V; skimmer voltage of 45 V.

The instrument gave a typical resolution of 18.000 Full Width at Half Maximum (FWHM) at *m/z* 118.0862 and 35,000 FWHM at *m/z* 922.0098. The instrument was calibrated and tuned as recommended by the manufacturer. To assure the desired mass resolution, continuous internal calibration was performed during analyses by using the signals at *m/z* 119.0362 (proton abstracted purine) and *m/z* 966.0007 (formate adduct).

The analytical samples were injected in auto-MS/MS acquisition mode to obtain information from the fragmentation of the main compounds. The collision energies were set at 10, 20, and 40 eV at 2.5 spectra s^−1^. 

Main compounds were detected automatically based on molecular feature detection, and the resulting peaks were filtered with a relative volume threshold of 0.2%, as well as those appearing in the solvent blank. Compounds detected with this algorithm were identified with METLIN database (URL http://metlin.scripps.edu (accessed on 29 April 2024)), HMDB (URL https://hmdb.ca/ (accessed on 29 April 2024)) and CAS SciFinder (URL https://www.cas.org/es-es/solutions/cas-scifinder-discovery-platform/cas-scifinder-n (accessed on 29 April 2024)).

#### 3.4.2. Quantification of Phenolic Compounds by LC−MS

For quantification of previously identified phenolic compounds, the sample extracts were analyzed by LC−TOF MS. MicroTOF system used for quantification worked in negative mode, with a mass range of 50–1000 m/z. Parameters of ESI−TOF were established as follows: capillary voltage, 4 kV; drying gas temperature, 190 °C; gas drying flow, 9 L min^−1^; nebulizing gas pressure, 2.0 bar; capillary voltage, −120 V; skimmer 1 voltage, −40 V; hexapole voltage, −23 V; hexapole radiofrequency, 100 Vpp; and skimmer 2 voltage, −22.5 V. External mass spectrometer calibration was performed with sodium formiate clusters (5 mM sodium hydroxide in water/2-propanol 1/1 (*v*/*v*), with 0.2% of formic) in quadratic þ high precision calibration (HPC) regression mode. The calibration solution was injected at the beginning of the run, and all the spectra were calibrated.

Regarding quantitative results, the phenolic compounds Hyty and Ty were quantified by the calibration curves obtained from their respective commercial standards. The other phenolic compounds, which had no commercial standards, were tentatively quantified with other compounds having similar or related structures. Hyty-Ac and glucoside were quantified using a Hyty calibration curve. The oleuropein standard was used to quantify compounds from the secoiridoid group. Table S2 shows the calibration parameters for the standards used in the quantification. The concentration of the compounds present in them was established by calculating the area of each individual compound and by interpolation on its corresponding calibration curve. Concentrations of compounds were expressed in µg of compounds per g of EVOO as the mean and standard deviation (SD) of two different injections from two different extracts (n = 4).

### 3.5. AChE Inhibition Assay

AChE inhibitory activity was measured using a spectrophotometric assay described by Ellman et al. [68] with slight modifications. First, in a 96-well plate, the AChE enzyme (10 mU mL^−1^) (dissolved in 50 mM Tris-HCl buffer, pH 8.0) was incubated with the DTNB (150 μM) and 12 μL aliquots of the corresponding EVOO extracts in a final reaction volume of 250 μL. In addition, the inhibition control (physostigmine), the standards Ol, Lut, and Hyty, and the positive control of AChE activity (substituting the extracts/standards by the solvent of the extracts: ethanol:water (1:1, *v*/*v*) were tested. After 15 min of incubation at 30 °C, the substrate (acetylthiocholine iodide 150 μM, ATCh) was added, and AChE activity was determined by measuring the increase in absorbance reading at 405 nm for 15 min at 30 °C in a spectrophotometer (BioTek Synergy, Agilent Technologies, Santa Clara, CA, USA).

The AChE inhibitory activity of the EVOO extracts was expressed as a percentage of inhibition with respect to the positive activity control, indicating the average value obtained by analyzing the 3 extraction replicates obtained from each EVOO extract and analyzing them in duplicate. IC_50_, the concentration of a standard bioactive compound capable of inhibiting 50% of AChE activity, was determined by measuring 5 different concentrations of the standard compound in the reaction medium and interpolating in the linear regression of the dosis-response curve.

### 3.6. COX-2 Inhibition Assay

The COX-2 assay was performed using the COX fluorescent inhibitor assay kit with slight modifications and measured spectrophotometrically (BioTek Synergy, Agilent Technologies, Santa Clara, CA, USA). EVOO extracts were diluted (1/200) in ethanol:water (1:1, *v*/*v*). Briefly, the assay was carried out in a 96-well plate, in which 10 μL of diluted EVOO extracts and 80 μL of the reaction mix (containing the cofactor, the probe, and the COX-2 enzyme) were added. After 2 min of incubation, the substrate (arachidonic acid) was added, and COX-2 activity was determined by measuring the increase in fluorescence (λEx = 535 nm/λEm = 587 nm) after 8 min at 37 °C. The COX-2 inhibitory activity of the EVOO extracts was expressed as a percentage of inhibition relative to the positive activity control (substituting the extracts/standards by the solvent of the extracts: ethanol:water (1:1, *v*/*v*). Also, celecoxib (a non-steroidal anti-inflammatory drug and a selective COX-2 inhibitor that acts by reducing pain and inflammation) and the standards Ol and Hyty were tested for their COX-2 inhibitory activity. EVOO extracts were analyzed in duplicate. IC_50_, the concentration of a standard bioactive compound capable of inhibiting 50% of COX-2 activity, was determined by measuring 5 different concentrations of the standard compound in the reaction medium and interpolating in the linear regression of the dosis-response curve.

### 3.7. Statistical Analysis

Quantification of EVOO phenolic compounds was assessed from four determinations, and results were expressed as mean and ± SD. The AChE and COX-2 inhibition assays were expressed as percentages of inhibition with respect to the positive control (Celecoxib), with 3 replicates of each extract and each replica analyzed twice (n = 6).

To study the correlation between the concentration of phenolic compounds and the neuroprotective and anti-inflammatory activity of EVOOs, Pearson’s correlation analysis was used. In addition, the similarities and differences among EVOOs were studied, and for this purpose, a comparative analysis between EVOOs was made according to genetic varieties and geographic situation (in terms of PDO). To evaluate the differences between EVOOs, the Kruskal-Wallis test was used, and to study the variability and the discriminatory power, PCA and Random Forest analyses were performed. Lastly, to comprehend differences between PDO, a Wilcoxon test was used. A difference was considered significant between groups when *p* values were less than 0.05 (95% confidence level). Data were treated as non-parametrical variables using the online software Metaboanalyst (https://www.metaboanalyst.ca/) and Statgraphics v.16.1 (Statgraphics Technologies, Inc., The Plains, Virginia, USA).

## 4. Conclusions

In this study, 15 samples of EVOO have been characterized in terms of phenolic compounds. Furthermore, their neuroprotective potential has been studied by enzyme activity inhibition essays (AChE and COX-2). All samples were able to inhibit AChE, with ranges between 20.7 and 86.8%, and COX-2 enzyme was inhibited in a similar way between samples, ranged 53–68%. Statistical analysis to correlate EVOO, neuroprotective potential, and PDO shows that the higher the phenolic compounds, the higher the neuroprotective action, except for lignans. Also, we observed statistically significant differences in the phenolic composition between PDOs, although more studies in this field are necessary.

The hypotheses in the scientific community about the beneficial properties of phenolic compounds in EVOO are becoming increasingly validated. In this study, it has been demonstrated that EVOOs have anti-inflammatory and neuroprotective action by in vitro assays, and a correlation among all quantified compounds and this biological action has been studied. For the first time, statistical analysis between phenolic compounds and EVOO’s PDO has been explored, revealing clear differences between the two PDO studies. Nevertheless, more studies and more varieties of EVOO are necessary to correlate the phenolic compounds, PDO, and biological defense they can provide. 

## Figures and Tables

**Table 1:** Concentration of the compounds tentatively identified in EVOO samples analyzed.

	Sample 1	Sample 2	Sample 3	Sample 4	Sample 5	Sample 6	Sample 7	Sample 8	Sample 9	Sample 10	Sample 11	Sample 12	Sample 13	Sample 14	Sample 15
**PDO**	PG	PG	MG	PG	PG	PG	PG	PG	MG	PG	PG	MG	MG	MG	PG
**Variety**	Hojiblanca	Picudo	Picual	Chorreao	Hojiblanca	Manzanillo	Picudo	Chorreao	Picual	Hojiblanca	Hojiblanca	Picual	Picual	Coupage	Picual
Concentration of the compounds expressed as µg of compounds g^−1^ of EVOO															
**DiHyGli**	1.4 ± 0.2	1.0 ± 0.1	0.67 ± 0.03	0.10 ± 0.01	0.13 ± 0.01	0.09 ± 0.01	0.25 ± 0.02	0.12 ± 0.01	0.25 ± 0.02	0.38 ± 0.04	0.47 ± 0.04	0.06 ± 0.01	0.04 ± 0.01	0.18 ± 0.01	0.5 ± 0.1
**ForP**	1.6 ± 0.2	7.8 ± 0.1	0.53 ± 0.04	0.010 ± 0.004	0.77 ± 0.03	0.08 ± 0.01	0.68 ± 0.04	2.07 ± 0.03	1.1 ± 0.2	0.67 ± 0.04	1.4 ± 0.1	0.009 ± 0.002	0.20 ± 0.02	0.31 ± 0.04	0.9 ± 0.1
**TMP-Ac**	2.4 ± 0.3	2.83 ± 0.2	0.35 ± 0.04	2.4 ± 0.1	1.04 ± 0.05	2.6 ± 0.1	1.7 ± 0.1	5.4 ± 0.3	2.0 ± 0.2	0.6 ± 0.1	0.57 ± 0.04	1.70 ± 0.04	0.54 ± 0.02	1.8 ± 0.1	0.8 ± 0.1
**DEDA-Ac**	0.5 ± 0.1	0.38 ± 0.05	0.88 ± 0.06	1.4 ± 0.1	0.08 ± 0.01	1.3 ± 0.1	0.9 ± 0.1	1.34 ± 0.04	0.83 ± 0.02	0.25 ± 0.04	0.22 ± 0.01	0.12 ± 0.02	0.19 ± 0.02	0.49 ± 0.04	0.49 ± 0.02
**Ac-Pin**	0.3 ± 0.0	0.50 ± 0.01	0.026 ± 0.004	0.068 ± 0.002	0.78 ± 0.02	0.12 ± 0.01	0.033 ± 0.004	0.164 ± 0.005	0.070 ± 0.003	0.18 ± 0.01	0.282 ± 0.004	0.20 ± 0.01	2.7 ± 0.01	0.82 ± 0.02	0.17 ± 0.01
**Api**	1.3 ± 0.1	1.56 ± 0.1	0.28 ± 0.02	0.70 ± 0.04	1.27 ± 0.03	0.62 ± 0.02	0.95 ± 0.04	0.13 ± 0.01	0.52 ± 0.05	1.0 ± 0.1	0.47 ± 0.03	0.64 ± 0.04	0.77 ± 0.01	0.8 ± 0.1	1.39 ± 0.03
**DEDA**	<LOQ	<LOQ	0.82 ± 0.04	0.41 ± 0.02	1.5 ± 0.1	0.8 ± 0.1	2.2 ± 0.1	5.1 ± 0.1	4 ± 1	2.9 ± 0.1	0.30 ± 0.02	0.39 ± 0.04	0.35 ± 0.05	0.5 ± 0.1	4.2 ± 0.2
**EA 1**	2.4 ± 0.1	3.2 ± 0.1	0.36 ± 0.02	0.77 ± 0.02	1.4 ± 0.3	1.2 ± 0.4	0.9 ± 0.1	0.7 ± 0.2	1.4 ± 0.1	1.1 ± 0.4	1.9 ± 0.1	1.4 ± 0.2	0.4 ± 0.1	1.2 ± 0.1	0.9 ± 0.1
**EA 2**	37.1 ± 1.0	28 ± 2	34 ± 2	51 ± 2	38 ± 2	21 ± 1	45 ± 3	56 ± 3	45 ± 1	64 ± 5	115 ± 3	79 ± 2	6.5 ± 0.3	26 ± 1	15.0 ± 0.3
**Hy-D-Li-Agl**	79 ± 3	63 ± 2	199 ± 4	387 ± 9	37 ± 2	341 ± 4	111 ± 1	232 ± 7	104 ± 3	55 ± 2	90 ± 4	43 ± 1	93 ± 3	83 ± 3	58 ± 1
**HyDec Ol Ag (I1)**	1.4 ± 0.2	0.9 ± 0.1	1.5 ± 0.1	3.0 ± 0.1	1.2 ± 0.1	2.2 ± 0.2	1.8 ± 0.1	2.4 ± 0.1	1.4 ± 0.1	5.4 ± 0.3	1.22 ± 0.04	0.61 ± 0.03	0.66 ± 0.04	1.1 ± 0.1	3.0 ± 0.3
**HyDec Ol Ag (I2)**	3.3 ± 0.4	3.9 ± 0.2	3.9 ± 0.2	4.9 ± 0.4	2.2 ± 0.3	8.3 ± 0.2	7.6 ± 0.2	14 ± 1	5.5 ± 0.5	1.8 ± 0.1	1.9 ± 0.1	2.1 ± 0.1	4.2 ± 0.2	4.7 ± 0.2	2.4 ± 0.1
**Hy-EA**	2.4 ± 0.4	4.2 ± 0.1	1.2 ± 0.1	0.28 ± 0.04	2.5 ± 0.2	1.49 ± 0.04	1.6 ± 0.1	2.2 ± 0.1	2.6 ± 0.3	0.7 ± 0.1	1.8 ± 0.1	1.56 ± 0.04	0.6 ± 0.1	2.3 ± 0.2	0.62 ± 0.03
**Hy-Ol-Ag 1**	0.9 ± 0.1	1.4 ± 0.2	2.8 ± 0.1	0.8 ± 0.1	0.4 ± 0.1	1.1 ± 0.1	0.6 ± 0.1	1.9 ± 0.1	5.2 ± 0.4	0.3 ± 0.1	1.1 ± 0.1	3.2 ± 0.3	0.09 ± 0.03	0.6 ± 0.1	0.9 ± 0.1
**Hy-Ol-Ag 2**	0.5 ± 0.1	1.2 ± 0.1	2.6 ± 0.2	0.6 ± 0.1	<LOD	0.8 ± 0.1	0.34 ± 0.04	1.7 ± 0.1	5.2 ± 0.5	<LOQ	0.5 ± 0.1	3.1 ± 0.1	0.04 ± 0.01	0.34 ± 0.04	0.42 ± 0.03
**Hy-Ol-Ag 3**	1.4 ± 0.2	2.2 ± 0.1	5.4 ± 0.3	1.5 ± 0.2	1.02 ± 0.03	4.1 ± 0.2	3.8 ± 0.1	4.2 ± 0.3	7 ± 1	1.6 ± 0.2	2.4 ± 0.1	3.3 ± 0.1	0.87 ± 0.02	2.8 ± 0.2	1.3 ± 0.1
**Hy-Ol-Ag 4**	0.7 ± 0.1	1.24 ± 0.04	0.07 ± 0.02	0.16 ± 0.01	0.36 ± 0.01	0.37 ± 0.03	0.77 ± 0.02	0.60 ± 0.05	0.7 ± 0.1	0.28 ± 0.04	0.31 ± 0.01	0.57 ± 0.04	0.16 ± 0.01	0.4 ± 0.1	0.16 ± 0.01
**Hyty**	2.1 ± 0.2	2.8 ± 0.1	6.5 ± 0.1	3.5 ± 0.1	2.3 ± 0.1	3.7 ± 0.2	7.7 ± 0.1	9.3 ± 0.2	12 ± 2	3.3 ± 0.2	7.1 ± 0.1	4.4 ± 0.2	3.9 ± 0.2	3.8 ± 0.1	5.6 ± 0.1
**Hyty-Ac**	<LOD	<LOD	0.93 ± 0.02	<LOD	0.02 ± 0.01	<LOD	<LOD	<LOD	0.12 ± 0.02	<LOD	0.118 ± 0.004	0.34 ± 0.03	1.1 ± 0.1	0.71 ± 0.03	0.54 ± 0.02
**Li-Ag 1**	39 ± 5	26 ± 2	4 ± 0.2	7 ± 1	7.5 ± 0.2	31 ± 3	8 ± 1	5.6 ± 0.3	58 ± 4	6 ± 1	21 ± 1	39 ± 2	0.23 ± 0.01	6.3 ± 0.2	7.1 ± 0.3
**Li-Ag 2**	17 ± 3	12 ± 1	9.2 ± 0.4	10.8 ± 0.4	8.8 ± 0.4	21 ± 2	15 ± 1	6.4 ± 0.3	33 ± 2	11 ± 1	8 ± 1	17 ± 0.5	0.47 ± 0.02	6.3 ± 0.2	6.5 ± 0.3
**Li-Ag 3**	10 ± 1	8.0 ± 0.4	8 ± 1	8.1 ± 0.5	6.2 ± 0.6	15 ± 1	10 ± 1	5 ± 1	16 ± 2	8 ± 1	9 ± 2	12 ± 1	0.14 ± 0.04	3.82 ± 0.04	4.2 ± 0.1
**Li-Ag 4**	54 ± 5	74 ± 2	246 ± 5	118 ± 4	45 ± 1	348 ± 4	127 ± 4	94 ± 2	219 ± 6	74 ± 1	74 ± 1	50 ± 3	14 ± 2	61 ± 1	49 ± 1
**Li-Ag 5**	4.5 ± 0.2	3.9 ± 0.1	33 ± 0.1	7.9 ± 0.7	4.7 ± 0.2	9 ± 1	7.5 ± 0.3	7.6 ± 0.2	9.6 ± 0.4	5.8 ± 0.3	6 ± 1	6.3 ± 0.2	1.12 ± 0.03	5.6 ± 0.2	<LOQ
**Li-Ag 6**	6.6 ± 0.2	5.3 ± 0.1	11.9 ± 0.4	13 ± 2	7.8 ± 0.3	21 ± 2	6.7 ± 0.3	13 ± 1	11 ± 1	10 ± 1	6.3 ± 0.4	9 ± 1	4.2 ± 0.3	10.9 ± 0.3	9.1 ± 0.3
**Li-Ag 7**	2.4 ± 0.1	1.9 ± 0.1	1.81 ± 0.04	2.8 ± 0.2	2.1 ± 0.1	3.0 ± 0.1	3.1 ± 0.2	2.2 ± 0.1	2.7 ± 0.3	2.6 ± 0.2	1.4 ± 0.2	2.3 ± 0.1	0.13 ± 0.02	1.58 ± 0.58	2.4 ± 0.1
**Lut**	2.3 ± 0.3	2.7 ± 0.1	1.00 ± 0.04	1.00 ± 0.03	2.34 ± 0.03	0.80 ± 0.03	1.0 ± 0.1	0.55 ± 0.02	1.9 ± 0.1	1.9 ± 0.1	1.10 ± 0.05	1.2 ± 0.1	2.14 ± 0.03	1.5 ± 0.1	2.8 ± 0.1
**Me-Ol-Ag 1**	<LOQ	<LOQ	1.6 ± 0.1	<LOQ	<LOQ	<LOQ	<LOQ	<LOQ	1.2 ± 0.2	0.7 ± 0.1	2.58 ± 0.05	0.4 ± 0.05	<LOQ	<LOQ	<LOQ
**Me-Ol-Ag 2**	<LOQ	<LOQ	0.7 ± 0.2	<LOQ	<LOQ	<LOQ	<LOQ	<LOQ	0.8 ± 0.1	0.11 ± 0.03	1.9 ± 0.1	0.20 ± 0.01	<LOQ	<LOQ	<LOQ
**Me-Ol-Ag 3**	<LOQ	<LOQ	1.2 ± 0.1	<LOQ	<LOQ	<LOQ	<LOQ	<LOQ	0.6 ± 0.1	<LOQ	1.3 ± 0.1	<LOQ	<LOQ	<LOQ	<LOQ
**Me-Ol-Ag 4**	<LOQ	0.32 ± 0.01	0.64 ± 0.2	0.16 ± 0.02	0.21 ± 0.03	<LOQ	0.14 ± 0.01	<LOQ	0.15 ± 0.04	0.29 ± 0.04	0.6 ± 0.1	0.07 ± 0.03	0.36 ± 0.04	0.32 ± 0.03	<LOQ
**Me-Ol-Ag 5**	0.30 ± 0.03	0.2 ± 0.01	24 ± 2	0.9 ± 0.1	<LOQ	0.39 ± 0.02	0.86 ± 0.03	<LOQ	3.7 ± 0.3	2.8 ± 0.1	5.5 ± 0.1	1.39 ± 0.04	1.3 ± 0.1	1.9 ± 0.1	0.24 ± 0.02
**Me-Ol-Ag 6**	0.13 ± 0.04	<LOQ	6.6 ± 0.3	0.6 ± 0.1	<LOQ	0.14 ± 0.01	0.41 ± 0.02	<LOQ	1.6 ± 0.1	1.7 ± 0.1	2.8 ± 0.1	0.71 ± 0.04	1.04 ± 0.04	1.3 ± 0.1	<LOQ
**Ol-Ag 1**	9.7 ± 0.5	17 ± 1	8 ± 1	6.2 ± 0.5	3.5 ± 0.2	7.7 ± 0.4	5.7 ± 0.4	5 ± 1	23 ± 3	4.8 ± 0.5	7.0 ± 0.3	2.6 ± 0.3	0.7 ± 0.1	3.4 ± 0.3	3.7 ± 0.1
**Ol-Ag 2**	82 ± 3	51 ± 3	777 ± 4	320 ± 7	46 ± 1	422 ± 5	193 ± 6	236 ± 9	569 ± 8	95 ± 2	131 ± 3	114 ± 3	84 ± 4	226 ± 6	122 ± 11
**Ol-Ag 3**	43 ± 1	33 ± 2	59 ± 1	57 ± 2	30 ± 2	64 ± 2	42 ± 2	44 ± 2	80 ± 4	30 ± 1	63 ± 1	41 ± 2	6.2 ± 0.1	43 ± 1	31 ± 1
**Ol-Ag 4**	6.0 ± 0.2	5.5 ± 0.3	5.3 ± 0.4	5.1 ± 0.2	3.6 ± 0.1	4.1 ± 0.1	4.6 ± 0.5	4.9 ± 0.1	7 ± 1	5.2 ± 0.5	4.2 ± 0.1	5.0 ± 0.2	0.82 ± 0.02	2.3 ± 0.1	4.1 ± 0.2
**Pin**	0.20 ± 0.01	0.25 ± 0.01	0.54 ± 0.02	0.16 ± 0.01	0.49 ± 0.01	0.18 ± 0.01	0.153 ± 0.004	0.252 ± 0.002	0.54 ± 0.02	0.27 ± 0.01	0.207 ± 0.01	0.66 ± 0.01	1.33 ± 0.03	0.79 ± 0.03	1.08 ± 0.02
**QuiAc**	0.08 ± 0.01	0.044 ± 0.003	3.2 ± 0.1	0.036 ± 0.001	0.019 ± 0.002	0.008 ± 0.002	<LOD	<LOD	0.9 ± 0.1	0.52 ± 0.03	1.2 ± 0.1	0.21 ± 0.01	<LOD	0.21 ± 0.03	0.78 ± 0.01
**Syr**	0.29 ± 0.01	0.30 ± 0.01	0.17 ± 0.01	0.19 ± 0.01	0.391 ± 0.002	0.228 ± 0.001	0.220 ± 0.005	0.127 ± 0.004	0.122 ± 0.003	0.40 ± 0.01	0.30 ± 0.01	0.22 ± 0.01	1.14 ± 0.03	0.48 ± 0.01	0.28 ± 0.01
**Tyr**	1.4 ± 0.1	1.3 ± 0.1	5.5 ± 0.3	3.4 ± 0.1	2.0 ± 0.1	2.4 ± 0.1	5.7 ± 0.1	10.8 ± 0.4	6 ± 1	3.3 ± 0.1	3.5 ± 0.1	2.2 ± 0.1	2.5 ± 0.2	1.8 ± 0.1	3.9 ± 0.1
**Van**	0.5 ± 0.1	0.65 ± 0.02	0.19 ± 0.01	0.25 ± 0.01	0.50 ± 0.02	0.26 ± 0.01	0.21 ± 0.02	0.19 ± 0.01	0.6 ± 0.1	0.54 ± 0.04	0.14 ± 0.01	0.24 ± 0.01	0.45 ± 0.03	0.39 ± 0.03	0.38 ± 0.01
**Total**	418	369	1471	1021	263	1341	619	775	1245	404	579	451	239	511	346

PDO, Protective Denomination of Origin; PG, *Poniente de Granada*; MG, *Montes de Granada*; Coupage: mix of picual, arbequino and hojiblanca; LOQ, Limit of quantification; LOD, Limit of Detection. Full name of abbreviations can be found in Table S1.

**Table 2:** AChE and COX-2 inhibition percentages (%) of EVOO extracts.

			AChE	COX-2
Sample *^a^*	PDO	Variety	% Inhibition	% Inhibition
1	PG	Hojiblanca	74.80 ± 7.12 b	63.26 ± 1.55 cde
2	PG	Picudo	75.04 ± 2.10 b	59.76 ± 2.37 ef
3	MG	Picual	73.09 ± 1.42 b	62.50 ± 1.00 cdef
4	PG	Chorreao	86.82 ± 0.36 a	63.63 ± 1.97 cde
5	PG	Hojiblanca	54.99 ± 5.05 e	54.58 ± 3.46 gh
6	PG	Manzanillo	87.21 ± 1.89 a	66.86 ± 0.84 bc
7	PG	Picudo	85.10 ± 1.89 a	64.42 ± 1.12 bcd
8	PG	Chorreao	77.85 ± 4.20 b	65.69 ± 4.29 bcd
9	MG	Picual	86.43 ± 1.83 a	68.48 ± 3.02 b
10	PG	Hojiblanca	63.65 ± 2.39 cd	53.04 ± 5.45 h
11	PG	Hojiblanca	58.89 ± 1.29 de	58.43 ± 4.45 fg
12	MG	Picual	67.00 ± 0.94 c	61.75 ± 1.34 def
13	MG	Picual	20.75 ± 0.49 g	58.89 ± 0.63 fg
14	MG	Coupage	46.02 ± 4.81 f	64.06 ± 1.48 cde
15	PG	Picual	45.63 ± 3.16 f	65.46 ± 1.02 bcd
Celecoxib	-	-	-	87.02 ± 1.10 a
Physostigmin	-	-	48.52 ± 1.69 f	-

PDO, Protective Denomination of Origin; PG, *Poniente de Granada*; MG, *Montes de Granada*; Coupage: mix of Picual, Arbequina, and Hojiblanca. Different letters in the same column indicate significant differences (*p* < 0.05, Kruskal-Wallis test) between the percentage inhibition of EVOO samples. *^a^* In the AChE inhibition assay, the bioactive compounds extracted from 0.150 g of EVOO and 0.025 µM of physostigmin were present in 250 μL of medium reaction containing 2.5 mU of AChE; in the COX-2 inhibition assay, the bioactive compounds extracted from 0.625 mg of EVOO and 0.050 µM of celecoxib were present in 100 μL of medium reaction.

**Table 3:** Results of Pearson’s correlation analysis of the quantified compounds and the percentage of inhibitory activity of AChE and COX-2.

	AChE		COX-2	
Compound	Correlation Coefficient	*p*-Value	Correlation Coefficient	*p*-Value
DEDA	0.7152	0.0027	0.6708	0.0062
Ac-Pin	-	-	−0.7969	0.0004
Hy-D-Li-Agl	-	-	0.5194	0.0473
HyDec Ol Ag 2	0.5902	0.0205	-	-
Hy-D-Ag 3	-	-	0.5637	0.0286
Hy Ol Ag 3	0.6107	0.0156	-	-
Li-Ag 2	-	-	0.6914	0.0043
Li-Ag 3	0.5823	0.0156	0.7813	0.0006
Li-Ag 4	0.5273	0.0434	0.6271	0.0124
Li-Ag 7	-	-	0.8122	0.0002
Ol-Ag 1	-	-	0.5577	0.0308
Ol-Ag 2	0.5422	0.0368	-	-
Ol-Ag 3	0.5457	0.0354	0.7138	0.0028
Ol-Ag 4	-	-	0.8066	0.0003
Pin	-	-	−0.8264	0.0001
Syr	-	-	−0.8343	0.0001

Full name of abbreviations can be found in Table S1. Statistically significant correlations are details (*p*-value < 0.05).

**Table 4:** Wilcoxon test results to evaluate differences between PDOs studied.

Compound	*p*-Value	Compound	*p*-Value
Api	0.011001	Me-Ol-Ag 1	0.006583
EA 1	0.005829	Me-Ol-Ag 2	0.002038
EA 2	0.015351	Me-Ol-Ag 3	0.010392
ForP	0.000522	Me-Ol-Ag 4	0.004056
Hy-D-Ol-Agl 1	9.92 × 10^−6^	Me-Ol-Ag 5	5.45 × 10^−7^
Hy-Ol-Ag 1	0.014051	Me-Ol-Ag 6	2.31 × 10^−6^
Hy-Ol-Ag 2	0.005130	Ol-Ag 1	0.010716
Hy-Ol-Ag 3	0.020644	Ol-Ag 2	0.011589
Hyty	0.002559	Pin	2.21 × 10^−14^
Hyty-Ac	9.96 × 10^−14^	QuiAc	0.000572
Li-Ag 2	0.000247	TMP-Ac	0.000322
Li-Ag 7	0.000192		

Full name of abbreviations can be found in Table S1.

## Data Availability

The original contributions presented in the study are included in the article, further inquiries can be directed to the corresponding author/s.

## Supplementary Materials

The following supporting information can be downloaded at: https://www.mdpi.com/article/10.3390/ijms25094878/s1.
