# Supplementary material for: Characterization of Phenolic Compounds in Extra Virgin Olive Oil from Granada (Spain) and Evaluation of Its Neuroprotective Action
Source: Int J Mol Sci. 2024 Apr 30;25(9):4878. doi: 10.3390/ijms25094878 (PMC11084348; doi:10.3390/ijms25094878)
Supplement: Supplementary file 1 [file ijms-25-04878-s001.zip › ijms-2953113-supplementary.pdf]

## SUPPLEMENTARY MATERIAL

**Table S1.** Tentatively identified compounds, abbreviation, retention time, m/z, molecular formula, error and fragments information.

| Compound | Abbreviation | TR (min) | m/z | Molecular formula | Error (ppm) | Fragments information |
|---|---|---|---|---|---|---|
| **Quinic acid** | QuiAc | 2.1 | 191.0568 | $C_7H_{12}O_6$ | -3.43 | 191.0572, 127.0408, 109.0282 |
| **3.4-Dihydroxyphenylglycol** | DiHyGli | 4.2 | 169.0514 | $C_8H_{10}O_4$ | -4.61 | 123.0453, 122.0372, 108.0235 |
| **Vanillic acid** | Van | 7.8 | 167.0359 | $C_8H_8O_4$ | -5.18 | 123.0455, 108.0218, 109.0296, 122.0365 |
| **Hydroxytyrosol** | Hyty | 8.6 | 153.0566 | $C_8H_{10}O_3$ | -5.71 | 123.0455, 153.056 |
| **3-(formylpropenyl) pentanoic acid** | ForP | 9.8 | 199.0622 | $C_9H_{12}O_5$ | -4.92 | 111.0819, 199.0615, 155.0711, 111.009 |
| **Tyrosol** | Ty | 9.9 | 137.061 | $C_8H_{10}O_2$ | -1.43 | |
| **Elenolic acid isomer 1** | EA 1 | 10.5 | 241.0729 | $C_{11}H_{14}O_6$ | -4.53 | 139.0040, 127.0402, 101.0247, 111.0091 |
| **Elenolic acid isomer 2** | EA 2 | 10.7 | 241.0732 | $C_{11}H_{14}O_6$ | -5.74 | 139.0037, 101.0244, 127.0400, 111.0088 |
| **Decarboxymethyl dialdehydic acid elenolic acid** | DEDA | 11.9 | 183.0672 | $C_9H_{12}O_4$ | -5.03 | |
| **Hydroxydecarboxymethyl oleuropein aglycone isomer 1** | HyDec Ol Ag 1 | 12.8 | 671.2358 | $C_{17}H_{20}O_7$ | -3.22 | 151.0405, 335.1142 |
| **Hydroxyoleuropein aglycone isomer 1** | Hy Ol Ag 1 | 13.5 | 393.1200 | $C_{19}H_{22}O_9$ | -2.06 | 151.0407, 123.0455, 139.0044 |
| **Trimethoxyphenylacetic acid** | TMP-Ac | 14.5 | 225.0782 | $C_{11}H_{14}O_5$ | -5.56 | 101.0245, 123.0450 |
| **Hydroxytyrosol acetate** | Hyty-Ac | 15.6 | 195.0669 | $C_{10}H_{12}O_4$ | -3.08 | 101.0244, 135.0453, 121.0292 |
| **Hydroxyelenolic acid** | Hy-EA | 17.4 | 257.068 | $C_{11}H_{14}O_7$ | -4.82 | 137.0606, 109.0658, 181.0505 |
| **Syringaresinol** | Syr | 17.5 | 417.1555 | $C_{22}H_{26}O_8$ | -0.02 | |
| **Pinoresinol** | Pin | 18.2 | 357.1344 | $C_{20}H_{22}O_6$ | -0.11 | |

| | | | | | | |
|---|---|---|---|---|---|---|
| **Hydroxydecarboxymethyl oleuropein aglycone isomer 2** | Hy-D-Ol-Agl 2 | 18.5 | 335.1149 | $C_{17}H_{20}O_7$ | -3.61 | 111.0816, 199.0617, 155.0717 |
| **Acetoxypinoresinol** | Ac-Pin | 18.6 | 415.1398 | $C_{22}H_{24}O_8$ | 0.1 | |
| **Hydroxyoleuropein aglycone isomer 1** | Hy-Ol-Ag 1 | 20.5 | 393.1206 | $C_{19}H_{22}O_9$ | -3.51 | 151.0396, 111.0083, 101.0244, 139.0036 |
| **Ligstroside aglycone isomer 1** | Li-Ag 1 | 20.9 | 361.1306 | $C_{19}H_{22}O_7$ | -3.65 | |
| **Methyloleuropein aglycone isomer 1** | Me-Ol-Ag 1 | 21.1 | 391.1409 | $C_{20}H_{24}O_8$ | -2.6 | |
| **Ligstroside aglycone isomer 2** | Li-Ag 2 | 21.3 | 361.1307 | $C_{19}H_{22}O_7$ | -3.93 | 101.0240, 291.0879, 111.0089 |
| **Methyloleuropein aglycone isomer 2** | Me-Ol-Ag 2 | 21.6 | 391.1411 | $C_{20}H_{24}O_8$ | -2.91 | 111.0095, 139.0036, 115.0401 |
| **Methyloleuropein aglycone isomer 3** | Me-Ol-Ag 3 | 21.8 | 391.1407 | $C_{20}H_{24}O_8$ | -2.27 | |
| **Hydroxydecarboxymethyl ligstroside aglycone** | Hy-D-Li-Agl | 22.0 | 319.1199 | $C_{17}H_{20}O_6$ | -3.33 | 199.0613, 111.0089, 111.0817, 155.0713, 181.0507 |
| **Ligstroside aglycone isomer 3** | Li-Ag 3 | 22.2 | 361.1314 | $C_{19}H_{22}O_7$ | -5.86 | 101.0246, 291.0879, 127.0403, 111.0088 |
| **Hydroxyoleuropein aglycone isomer 2** | Hy-Ol-Ag 2 | 22.3 | 393.1206 | $C_{19}H_{22}O_9$ | -3.58 | |
| **Ligstroside aglycone isomer 4** | Li-Ag 4 | 22.5 | 361.131 | $C_{19}H_{22}O_7$ | -4.4 | 101.0247, 291.0886, 127.0405, 111.0090, 292.0917 |
| **Methyloleuropein aglycone isomer 4** | Me-Ol-Ag 4 | 22.6 | 391.1407 | $C_{20}H_{24}O_8$ | -2.05 | |
| **Oleuropein aglycone isomer 1** | Ol-Ag 1 | 23.6 | 751.2268 | $C_{19}H_{20}O_8$ | -3.48 | 111.0090, 149.0245, 139.0405, 101.0246 |
| **Hydroxyoleuropein aglycone isomer 3** | Hy-D-Ag 3 | 24.8 | 393.1206 | $C_{19}H_{22}O_9$ | -3.59 | |
| **Oleuropein aglycone isomer 2** | Ol-Ag 2 | 25.0 | 377.1256 | $C_{19}H_{22}O_8$ | -3.65 | 111.0090, 149.0245, 139.0405, 101.0246 |
| **Luteolin** | Lut | 25.5 | 285.0421 | $C_{15}H_{10}O_6$ | -5.39 | 133.0293, 285.0402, 151.0033 |
| **Ligstroside aglycone isomer 5** | Li-Ag 5 | 26.0 | 361.1308 | $C_{19}H_{22}O_7$ | -3.79 | 101.0246, 291.0878, 127.0400, 111.0090 |

| | | | | | | |
|---|---|---|---|---|---|---|
| **Methyloleuropein aglycone isomer 5** | Me-Ol-Ag 5 | 26.1 | 391.1414 | $C_{20}H_{24}O_8$ | -3.81 | 111.0086, 139.0036, 139.0399, 115.0397, 141.0556 |
| **Oleuropein aglycone isomer 3** | Ol-Ag 3 | 26.2 | 377.1257 | $C_{19}H_{22}O_8$ | -3.75 | 111.009, 101.0247, 139.0039, 149.0245, 139.0401 |
| **Ligstroside aglycone isomer 6** | Li-Ag 6 | 26.3 | 361.1305 | $C_{19}H_{22}O_7$ | -3.05 | 101.0249, 291.0878, 127.0404, 111.0091, 292.0910 |
| **Elenolic decarboxymethyl dialdehydic acid acetal** | DEDA-Ac | 26.4 | 365.1618 | $C_{19}H_{26}O_7$ | -3.28 | |
| **Methyloleuropein aglycone isomer 6** | Me-Ol-Ag 6 | 26.5 | 391.1409 | $C_{20}H_{24}O_8$ | -2.4 | 111.0086, 139.0036, 115.0397, 141.0556 |
| **Apigenin** | Api | 26.7 | 269.0466 | $C_{15}H_{10}O_5$ | -3.71 | 117.0348, 269.0458, 151.0038, 149.0245 |
| **Oleuropein aglycone isomer 4** | Ol-Ag 4 | 26.7 | 377.1257 | $C_{19}H_{22}O_8$ | -3.72 | 111.0086, 139.0402, 139.0036, 101.0245, 149.0243 |
| **Ligstroside aglycone isomer 7** | Li-Ag 7 | 27.0 | 361.1311 | $C_{19}H_{22}O_7$ | -5.04 | |

**Table S2**. Calibration parameters for standards used in the quantification.

| Compound | Calibration range (μg $mL^{-1}$) | Calibration curve | $R^2$ | LOD (μg $mL^{-1}$) | LOQ (μg $mL^{-1}$) |
|---|---|---|---|---|---|
| BenAc | 1–40 | $y = -59.9 x^2 + 7467.6 x + 11136$ | 0.9962 | 0.55 | 1.64 |
| Hyty | 1–150 | $y = -242 x^2 + 97878 x + 80667$ | 0.9987 | 0.07 | 0.20 |
| Ty | 1–40 | $y = 74.6 x^2 + 32965 x - 10370$ | 0.9989 | 0.14 | 0.42 |
| Lut | 1–40 | $y = -3209.1 x^2 + 355617 x + 401034$ | 0.9969 | 0.02 | 0.06 |
| Ol | 1–150 | $y = -237.4 x^2 + 74636 x + 264040$ | 0.9943 | 0.05 | 0.16 |
| Pin | 1–40 | $y = -661.7 x^2 + 104606 x + 5319.7$ | 0.9990 | 0.02 | 0.06 |
| QuinAc | 1–40 | $y = -417.1 x^2 + 123852 x + 21480$ | 0.9963 | 0.04 | 0.12 |
| Van | 1–40 | $y = -607.2 x^2 + 64345 x + 39052$ | 0.9960 | 0.06 | 0.19 |

*BenAc, benzylic acid; Hyty, hydroxytyrosol; Ty, tyrosol; Lut, luteoline; Ol, oleuropeine; Pin, pinoresinol; QuinAc, quinic acid; Van, vanillic acid; LOD, limit of detection; LOQ, limit of quantification.*
